# Identification of Staphylococcus aureus Factors Required for Pathogenicity and Growth in Human Blood

**DOI:** 10.1128/IAI.00337-17

**Published:** 2017-10-18

**Authors:** John Connolly, Emma Boldock, Lynne R. Prince, Stephen A. Renshaw, Moira K. Whyte, Simon J. Foster

**Affiliations:** a Department of Molecular Biology and Biotechnology, University of Sheffield, Sheffield, United Kingdom; b Florey Institute, University of Sheffield, Sheffield, United Kingdom; c Department of Infection, Immunity and Cardiovascular Disease, University of Sheffield, Sheffield, United Kingdom; d Bateson Centre, University of Sheffield, Sheffield, United Kingdom; Albert Einstein College of Medicine

**Keywords:** Staphylococcus aureus, blood, nucleotide

## Abstract

Staphylococcus aureus is a human commensal but also has devastating potential as an opportunistic pathogen. S. aureus bacteremia is often associated with an adverse outcome. To identify potential targets for novel control approaches, we have identified S. aureus components that are required for growth in human blood. An ordered transposon mutant library was screened, and 9 genes involved specifically in hemolysis or growth on human blood agar were identified by comparing the mutants to the parental strain. Three genes (*purA*, *purB*, and *pabA*) were subsequently found to be required for pathogenesis in the zebrafish embryo infection model. The *pabA* growth defect was specific to the red blood cell component of human blood, showing no difference from the parental strain in growth in human serum, human plasma, or sheep or horse blood. PabA is required in the tetrahydrofolate (THF) biosynthesis pathway. The *pabA* growth defect was found to be due to a combination of loss of THF-dependent dTMP production by the ThyA enzyme and increased demand for pyrimidines in human blood. Our work highlights *pabA* and the pyrimidine salvage pathway as potential targets for novel therapeutics and suggests a previously undefined role for a human blood factor in the activity of sulfonamide antibiotics.

## INTRODUCTION

The pathogenicity of the Gram-positive bacterium Staphylococcus aureus requires a multitude of virulence factors that are intricately coordinated and regulated (1, 2). In addition to the more “classic” virulence factors, such as pore-forming toxins and superantigens, fundamental metabolic processes of bacteria are also recognized as a prerequisite for disease. Indeed, the majority of antibiotics act by disrupting essential metabolic processes (3). However, pathogens, including S. aureus, have adapted so as to resist such insults by switching off, or severely reducing, the activity of aspects of metabolism in order to persist in the presence of antibiotics (4, 5).

Microbial fitness during pathogenesis requires efficient utilization of available nutrients. Although the mammalian host is nutrient rich, many nutrients are sequestered as a means of inhibiting pathogen growth, a concept referred to as “nutritional immunity” (6). Strategies for overcoming the nutrient-limited environment *in vivo* have been well described for S. aureus and other bacteria; they include the upregulation of peptide or amino acid transport mechanisms (7) and of proteins that enable the acquisition of nutrients sequestered by the host (8, 9). *De novo* biosynthetic pathways are also required to produce essential products not readily available in the environment. Nucleotide biosynthetic pathways have been identified as critical for the proliferation of Gram-positive pathogens on human blood (10), yet detailed studies of the growth requirements of S. aureus are lacking.

To support studies on S. aureus, the Nebraska transposon mutant library (NTML) was recently constructed in the community-acquired methicillin-resistant S. aureus (CA-MRSA) USA300 JE2 strain, deposited in the Network on Antimicrobial Resistance in S. aureus (NARSA) strain repository, and made freely available to registered users (11). This library was created using the mariner-based transposon (bursa aurealis) and employing the same methodology as that of Bae and colleagues (12). To date, the NTML has been used to carry out diverse screens to identify genes involved in S. aureus antibiotic persistence *in vitro* (13), altered hemolytic activity on rabbit blood agar (11, 14), polymicrobial interactions (15), and hyaluronidase activity (16).

A comprehensive approach to identify genes involved in the growth of S. aureus on human blood was undertaken using the NTML. The genes were then further characterized to analyze their potential roles in human infection. We show that purine biosynthesis is indispensable for growth on human blood and *in vivo* pathogenicity, using a zebrafish embryo model. In addition, a gene involved in tetrahydrofolate (THF) biosynthesis, *pabA*, was identified as being required for virulence *in vivo* and was unable to grow specifically on human blood. The relationship between human blood, a folate-poor environment, and S. aureus pyrimidine salvage pathways was further elucidated.

## RESULTS

### Screening of an S. aureus Tn library for growth defects on human blood.

The NTML was screened to define gene disruptions leading to alterations in growth and/or hemolysis on agar containing human blood as the only nutrient source (see Materials and Methods). The library was also screened on bovine serum agar and 5% (vol/vol) sheep blood with a Columbia agar base as comparators, in order to determine traits specific to human blood (data not shown). The transposon (Tn) insert for each strain identified in the screen was transduced back into the parent strain (S. aureus JE2), and transductants were rescreened in order to establish that the mutant phenotype was associated with each Tn insertion. Fifteen transductants maintained the altered phenotype, and nine of these (the *purB*, *purA*, *pabA*, *atl*, *murQ*, *araC*, *mecA*, *odhB*, and *lipA* transductants) were selected for further study (Table 1). The remaining six strains had transposon disruptions in genes expected to produce an altered phenotype when grown on human blood agar (*agrA*, *agrB*, *agrC*, *hla*, *saeR*, and *saeS*), confirming the ability of the screen to identify specific phenotypes.

**TABLE 1:** Tn library mutants identified as having an altered phenotype on human blood agar

Category^*a*^	Protein ID	NARSA ID	Protein name	Growth phenotype in:		Hemolysis phenotype in:	
				Human blood	Rabbit blood	5% human blood + Columbia agar	5% sheep blood + Columbia agar
A1	SAUSA300_1889	NE522	Adenylosuccinate lyase (PurB)	Reduced growth	Reduced growth	Increased hemolysis	Reduced hemolysis
	SAUSA300_0017	NE529	Adenylosuccinate synthetase (PurA)	Reduced growth	Reduced growth	Increased hemolysis	Slightly increased hemolysis
	SAUSA300_0698	NE821	*para*-Aminobenzoate synthase, glutamine amidotransferase, component II (PabA)	Highly reduced growth	Slightly reduced growth	—^*b*^	—
A2	SAUSA300_0955	NE460	Autolysin (Atl)	Opaque colony	Opaque colony	Increased hemolysis	—
	SAUSA300_0193	NE1253	*N*-Acetylmuramic acid-6-phosphate etherase (MurQ)	—	—	Increased hemolysis	Increased hemolysis
	SAUSA300_2326	NE1304	Transcription regulatory protein (AraC)	—	—	Reduced hemolysis	—
	SAUSA300_0899	NE1315	Adaptor protein (MecA)	—	—	Reduced hemolysis	Slightly reduced hemolysis
	SAUSA300_1305	NE1391	Dihydrolipoamide succinyltransferase (OdhB)	Slightly reduced growth	Slightly reduced growth	Increased hemolysis	Increased hemolysis
	SAUSA300_0320	NE1775	Triacylglycerol lipase (LipA)	Slightly reduced growth	—	Increased hemolysis	—
B	SAUSA300_1989	NE95	Accessory gene regulator protein B (AgrB)	—	—	—	Reduced hemolysis
	SAUSA300_1991	NE873	Accessory gene regulator protein C (AgrC)	—	—	Slightly reduced hemolysis	No hemolysis
	SAUSA300_0690	NE1296	Sensor histidine kinase (SaeS)	—	—	—	No hemolysis
	SAUSA300_1058	NE1354	Alpha-hemolysin (Hla)	—	—	—	No hemolysis
	SAUSA300_1992	NE1532	Accessory gene regulator protein A (AgrA)	—	—	Reduced hemolysis	No hemolysis
	SAUSA300_0691	NE1622	DNA-binding response regulator (SaeR)	—	—	—	No hemolysis

a A1, strains with a defect in growth on human blood agar, which were investigated further; A2, strains with altered hemolysis on human blood agar, which were investigated further; B, strains expected to show a hemolysis phenotype, which were not explored further.

b —, no difference from the control strain, JE2.

### Phenotypic characterization of growth-defective mutants *in vivo*.

**FIG 1:**
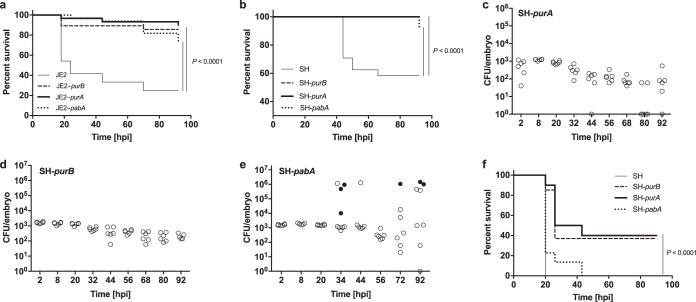
*In vivo* characterization of S. aureus strains in the zebrafish embryo model of infection with reduced growth in human blood *in vitro*. (a) Survival curves of fish injected with wild-type S. aureus JE2 or with JE2-*purA*, JE2-*purB*, or JE2-*pabA* (1,500 CFU each). (b) Survival curves of fish injected with wild-type S. aureus SH1000 (SH) or with SH1000 *purA*, SH1000 *purB*, or SH1000 *pabA* (1,500 CFU each). (c to e) Growth of S. aureus mutants within embryos after injection with 1,500 CFU of SH1000 *purA* (c), SH1000 *purB* (d), or SH1000 *pabA* (e). Open circles, live embryos; filled circles, dead embryos. (f) Survival curves of *pu.1* knockdown fish injected with wild-type S. aureus SH1000, SH1000 *purA*, SH1000 *purB*, or SH1000 *pabA* (1,500 CFU).

In order to define genes for further study, the pathogenicity of the nine transduced strains in the JE2 background was assessed using the zebrafish embryo model of systemic S. aureus infection (17). The *atl*, *murQ*, *araC*, *mecA*, *odhB*, and *lipA* transductants did not show altered killing in this model (see Fig. S1a and b in the supplemental material). However, three of the strains, harboring Tn inserts in the *purA*, *purB*, and *pabA* genes (referred to here as JE2-*purA*, JE2-*purB*, and JE2-*pabA*), showed significant attenuation in the zebrafish model (*P* < 0.0001) (Fig. 1a). To confirm that the reduced pathogenicity was not strain specific, Tn inserts containing the *purA*, *purB*, and *pabA* genes were transduced into another strain background, S. aureus SH1000. These strains (referred to here as SH-*purA*, SH-*purB*, and SH-*pabA*) also showed significant attenuation in the zebrafish embryo model (*P* < 0.0001) (Fig. 1b). *In vivo* growth analysis demonstrated that SH-*purA* and SH-*purB* were unable to replicate within zebrafish embryos, and the numbers of bacteria recovered were lower than the inoculated dose (Fig. 1c and d). This is in stark contrast to the bacterial kinetics observed when the parental S. aureus strain is injected at the same dose as that published previously (Fig. S1c) (17). SH-*pabA* retained limited capacity to replicate and to cause host death in the zebrafish model (Fig. 1e). Using a knockdown approach to deplete zebrafish myeloid cells (morpholino-mediated *pu.1* knockdown), SH-*pabA* was restored to a level of virulence similar to that of the parental strain, but with a slight temporal delay (Fig. 1f). By 20 h postinfection (hpi), all embryos injected with the parent strain, and 80% of SH-*pabA*-injected embryos, had succumbed. The remaining SH-*pabA*-injected embryos died over the following 24 h. In myeloid cell-depleted zebrafish, SH-*purA* and SH-*purB* caused the death of approximately two-thirds of subjects injected, significantly less than the parent strain (*P* < 0.0001). The *pu.1* knockdown approach causes a temporary delay in phagocytic cell development, and as expected, no further host death was observed after 40 h, a time point at which phagocyte production would recover (18, 19).

### Purine biosynthesis is required for growth in blood.

**FIG 2:**
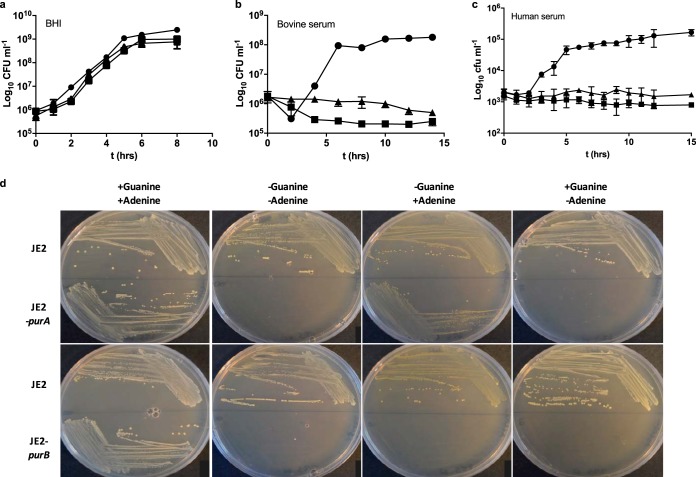
The S. aureus *purA* and *purB* mutants require exogenous purines for growth. (a to c) Strains were grown in BHI (a), bovine serum (b), or human serum (c). Data are from three independent repeats; error bars represent standard errors. Circles, JE2; squares, JE2-*purB*; triangles, JE2-*purA*. (d) Growth of strains on CDM agar plates with or without adenine (20 μg ml^−1^) and guanine (20 μg ml^−1^) after 24 h of aerobic incubation at 37°C.

Analysis of the *purA* and *purB* genes (20, 21) demonstrated that *purA* and *purB* code for enzymes in the purine biosynthesis pathways (adenylosuccinate synthase and adenylosuccinate lyase, respectively) (see Fig. S2 in the supplemental material). *In vitro*, JE2-*purA* and JE2-*purB* showed reduced growth on human blood and bovine serum agar plates but growth similar to that of the parent strain on 5% (vol/vol) sheep blood, which contained a rich nutrient base (data not shown). Growth assays of JE2-*purA* and JE2-*purB* were also conducted in liquid media (brain heart infusion [BHI], bovine serum, or human serum) (Fig. 2a to c). Growth was comparable to that of the parent only in the nutrient-rich BHI medium, matching that seen in the initial NTML screen. This finding suggested that the reduced-growth phenotype was due to a requirement for a nutrient not readily available in human blood or in human or bovine serum. Analysis of the purine biosynthesis pathway suggested that both strains should require adenine for growth, while in addition to adenine, JE2-*purB* should also require guanine (or inosine). Chemically defined medium (CDM) analysis confirmed that the growth of JE2-*purA* was dependent on the presence of adenine, while the growth of JE2-*purB* was dependent on adenine and guanine (Table 2; Fig. 2d). The addition of 20 μg ml^−1^ adenine and 20 μg ml^−1^ inosine restored the growth of each *pur* mutant, to levels similar to that obtained for the parent strain (data not shown). Biochemical complementation of the *purA* and *purB* mutants was not successful in the zebrafish infection model, likely due to poor diffusion of nucleobases into zebrafish embryos (data not shown). The importance of purine biosynthesis pathway enzymes in disease has been well characterized (22, 23).

**TABLE 2:** Growth analysis of JE2-*purA* and JE2-*purB* on solid media in the presence or absence of adenine and guanine

Strain	Growth^*a*^ on chemically defined medium			
	With adenine and guanine^*b*^	Without adenine or guanine	With adenine, without guanine	Without adenine, with guanine
JE2	+	+	+	+
*purB* mutant	+	−	−	−
*purA* mutant	+	−	+	−

a +, growth; −, no growth.

b The concentration of adenine or guanine, when present, was 20 μg ml^−1^.

### *pabA* is required for virulence in the murine sepsis model and for growth in human blood.

**FIG 3:**
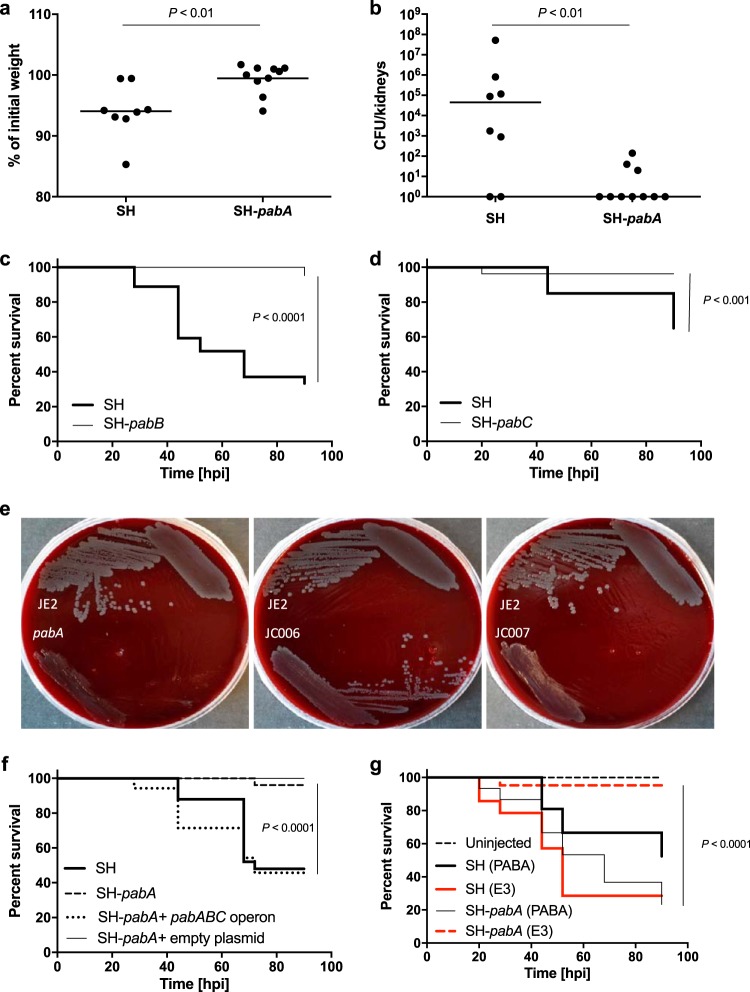
The *pabABC* operon is required for pathogenesis. (a and b) Female BALB/c mice (*n* = 10) were injected i.v. with 2 × 10^7^ CFU S. aureus SH1000 (SH) or 4 × 10^7^ CFU S. aureus SH1000 *pabA* (SH-*pabA*). Weight loss (a) and CFU counts in the kidneys (b) were measured after 3 days. (c) Survival curves of fish injected with S. aureus SH1000 (1,500 CFU) or S. aureus SH1000 *pabB*. (d) Survival curves of fish injected with S. aureus SH1000 (1,500 CFU) or S. aureus SH1000 *pabC*. (e) Growth of the parent strain (JE2), the *pabA* mutant, the genetically complemented *pabA* strain (with pJC002 integrated) (strain JC006), or a control integrated strain (with the empty pKASBAR plasmid in the *pabA* mutant) (strain JC007) on unsupplemented human blood agar (30%, vol/vol). Plates were incubated aerobically at 37°C for 48 h. (f) Survival curves of fish injected with 1,500 CFU of S. aureus SH1000, S. aureus SH1000 *pabA*, S. aureus SH1000 *pabA* plus the *pabABC* operon (with pJC002 integrated) (strain JC010), or S. aureus SH1000 *pabA* with an empty plasmid only (pKASBAR) (strain JC011). (g) Survival curves of fish injected with 1,500 CFU of S. aureus SH1000 or S. aureus SH1000 *pabA* followed by immediate immersion in either unsupplemented E3 medium (red) or E3 medium supplemented with 7 μg ml^−1^ PABA (black). Uninjected fish were included as controls under each condition.

In a mouse sepsis model, mice injected with S. aureus SH-*pabA* (4 × 10^7^ CFU) lost significantly less weight than those receiving the parent strain (2 × 10^7^ CFU) (Fig. 3a). Bacterial numbers were also significantly lower in kidneys harvested from mice injected with SH-*pabA* (Fig. 3b) (*P* < 0.01).

The *pabA* Tn mutant was found to have a unique growth phenotype in the initial screen. Growth was highly reduced on 30% (vol/vol) human blood but only slightly reduced on 30% (vol/vol) rabbit blood (Table 1). However, the *pabA* mutant grew well on both sheep and horse blood agar (30% [vol/vol]), demonstrating that the phenotype was species specific (data not shown). In addition, the *pabA* mutant demonstrated good growth on 50% (vol/vol) human serum or plasma agar (see Fig. S3 in the supplemental material). To ascertain if the amount of human plasma in 30% (vol/vol) whole blood agar was too small to support growth, the *pabA* mutant was compared to the parent strain on agar in which the plasma concentration was increased up to 50% (vol/vol). At the lower concentrations of 10% (vol/vol) and 15% (vol/vol) (15% being the approximate concentration of plasma in 30% [vol/vol] blood agar), the growth of the *pabA* mutant was poor but comparable to that of JE2. Therefore, the reduced growth of the *pabA* mutant on human blood was not a result of lower plasma levels in human blood agar (data not shown).

PabA is an enzyme required for tetrahydrofolate (THF) synthesis (*para*-aminobenzoate synthetase component II) (20), and *pabA* is found in an operon with *pabB* and *pabC*, which is responsible for the synthesis of the folate pathway intermediate 4-aminobenzoic acid (*para*-aminobenzoic acid [PABA]) (20). Strains from the NTML harboring a Tn disrupting *pabB* or *pabC* were transduced into the SH1000 background and were also found to be attenuated in the zebrafish infection model (Fig. 3c and d) (*P* < 0.001). Genetic complementation of the *pab* operon restored JE2-*pabA* growth on human blood (Fig. 3e) and SH-*pabA* virulence in the zebrafish model (Fig. 3f).

Reduced growth on human blood could be due to a lack of nutrients that are required by a strain lacking THF. The end product of the folate pathway, THF, acts as a single-carbon donor/acceptor in glycine/serine interconversion, vitamin B_5_ synthesis, methionine synthesis, purine synthesis, *N*-formylmethionine-tRNA charging, glycine cleavage, and dTMP synthesis (see Fig. S4 in the supplemental material). To further characterize *pabA*, different media were used to interrogate the mechanism underpinning the lack of growth on human blood. With liquid culture, the growth of the *pabA* mutant was comparable to that of the wild type in BHI, bovine serum, and human serum (data not shown), suggesting that the reduced-growth phenotype was specific to blood. When CDM lacking purines, serine, and glycine was used as the base, only the addition of purines, serine, and glycine together could restore the growth yield of the mutant to parental levels (as measured by the maximum optical density at 600 nm [OD_600_] reached) (data not shown). Biochemical complementation with the same supplements did not restore the growth of the *pabA* mutant on human blood, nor did the addition of folic acid. However, the addition of PABA fully complemented growth (see Fig. S5a in the supplemental material), as would be expected based on similar work done with Lactococcus lactis (24). Immersion of SH-*pabA*-injected zebrafish embryos in E3 medium containing PABA restored virulence *in vivo* (Fig. 3g) (*P* < 0.0001).

### Pyrimidine salvage pathways are required to bypass *pabA*.

**FIG 4:**
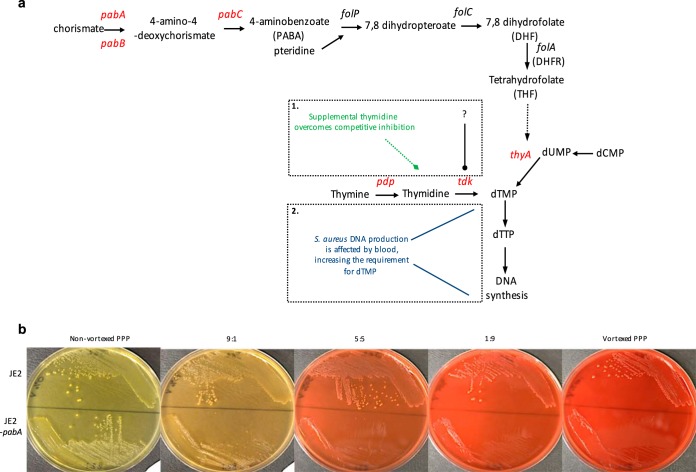
Folate biosynthesis pathway and effect of lysed RBCs on the growth of S. aureus *pabA*. (a) The folate biosynthesis and pyrimidine nucleotide salvage pathways (20, 21). Possible hypotheses for poor *pabA* growth on human blood are shown: either S. aureus Tdk is the target of competitive inhibition by human blood (frame 1) or increased dTMP demand necessitates supplemental thymidine in S. aureus (frame 2). (b) Growth of S. aureus JE2 or JE2-*pabA* on nonvortexed human PPP or with a decreasing ratio of vortexed to nonvortexed agar. Plates were incubated aerobically at 37°C for 48 h.

dTMP is synthesized via a THF-dependent route, or via an alternative nucleotide salvage pathway requiring thymine or thymidine (Fig. 4a). A combination of glycine, serine, and purines could not restore the growth of the *pabA* mutant on human blood; however, the addition of pyrimidines (thymine) supported growth to the extent of the parent strain, JE2 (Fig. S5a in the supplemental material). The crucial role of pyrimidines in bacterial survival under conditions of folate deprivation has been reported previously (25, 26). Neither pyrimidines nor folic acid could restore the pathogenicity of the *pabA* mutant in the zebrafish embryo model (data not shown).

Double mutants defective in *pabA* and one of the pyrimidine nucleotide salvage pathway genes—*pdp*, *tdk*, or the thymidine transporter gene, *nupC*—were constructed in order to assess the roles of these genes in *pabA* growth. The growth of all three double mutants was reduced on human blood but could be complemented with PABA (Fig. S5b in the supplemental material). The addition of thymine or thymidine to blood could complement all the mutants except for the *pabA tdk* double mutant. This highlighted the fact that pyrimidine salvage pathways are required to bypass the deficit of *pabA*. If an inhibitory factor in blood was responsible for preventing the growth of the *pabA* mutant, Tdk was the likely target. Unexpectedly, the *pabA pdp* double mutant was complemented by thymine, and the *pabA nupC* mutant was complemented by thymidine. This suggests that thymine can be converted to thymidine independently of Pdp and that an alternative thymidine transporter to NupC is available in S. aureus. Two remaining putative pyrimidine transporters have been identified in S. aureus and have not yet been investigated (27).

### Investigating a nucleotide salvage pathway inhibitory component in human blood.

The nucleotide salvage pathway appears to provide enough dTMP (later converted to dTTP) for the DNA synthesis and growth of the *pabA* mutant on human plasma or serum, but not on human blood, unless thymine or thymidine is added. This suggested that either a factor in whole blood competitively inhibits the nucleotide salvage pathway enzymes or growth on human blood leads to an increased requirement for dTMP, which cannot be met without increasing the thymine or thymidine concentration (Fig. 4a). To hone in on an inhibitory factor, different components of blood were assessed for their abilities to replicate the poor-growth phenotype of the *pabA* mutant seen on whole human blood. The growth of JE2-*pabA* was comparable to that of JE2 on platelet-rich plasma (PRP) and on PRP that had been vortexed to disrupt platelets (data not shown). Similarly, the growth of the parent strain and that of the mutant were equivalent when white blood cells (WBCs), either intact or lysed, were added to platelet-poor plasma (PPP). Vortexing of whole human blood followed by centrifugation produces red, rather than straw-colored, plasma, indicating red blood cell (RBC) lysis. Plasma from vortexed blood was mixed with PPP to give a 9:1 ratio of nonvortexed to vortexed plasma, decreasing incrementally to a ratio of 1:9. At the lowest ratio of nonvortexed to vortexed plasma, the growth of JE2-*pabA* was greatly reduced (Fig. 4b). This suggested that there is a potent inhibitor of the growth of the *pabA* mutant in the RBC component of human blood.

Hemoglobin or heme was deemed a likely candidate for the inhibitory factor. Hemoglobin, a complex of four heme groups, is the most abundant hemoprotein in humans. Heme is an iron-containing ring structure, and usage of heme as an iron source can be toxic to bacteria due to its active redox potential (28). Although the underlying mechanisms are not fully understood, it has been reported that heme-induced monooxygenase-like activity can cause direct DNA damage (28, 29). In S. aureus, heme is extracted from hemoglobin and is transported into the cell by the iron-regulated surface determinant (Isd) system (30). Toxicity induced by the liberation of iron from heme by S. aureus is reduced by the two-component heme-regulated transporter (*hrtAB*). Heme is also transported into S. aureus by the ABC transporter HtsABC, which requires the extraction of heme from hemoglobin by the Isd system (30). Both transport systems are upregulated under low Fe conditions by alleviation of the negative regulator Fur. However, supplementation of human blood agar with an alternative Fe source (ammonium ferrous sulfate) did not support the growth of JE2-*pabA* on human blood (data not shown). In addition, lyophilized bovine hemin, bovine hemoglobin, and human hemoglobin failed to prevent the growth of the *pabA* mutant on plasma (data not shown).

### S. aureus growth in human blood requires increased levels of pyrimidines.

**FIG 5:**
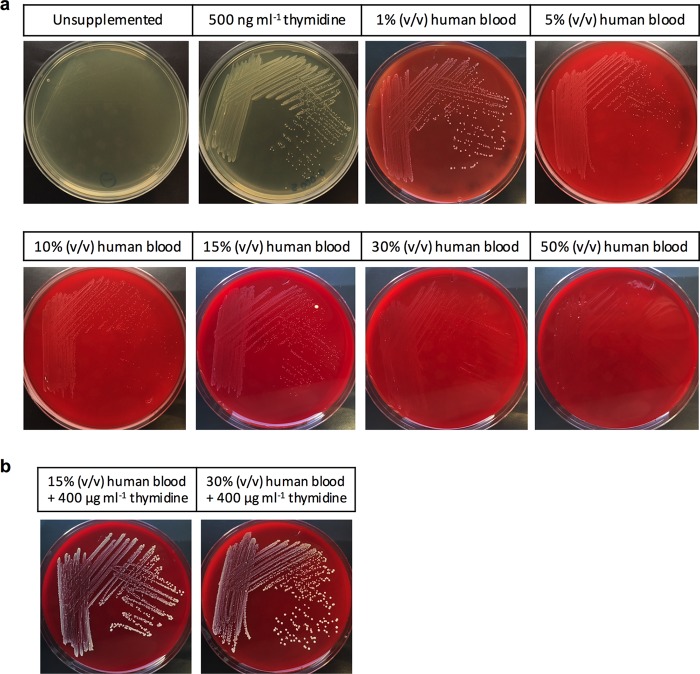
Increased concentrations of thymidine are required for S. aureus growth on human blood. (a) Growth of S. aureus SH-*thyA* on MH agar. The medium either was left unsupplemented (top left) or contained a permissive concentration of thymidine (500 ng ml^−1^). Increasing concentrations of human blood, ranging from 1 to 50% (vol/vol), were added to the MH agar base, which contained thymidine (500 ng ml^−1^). Plates were incubated aerobically at 37°C for 24 h. (b) At concentrations of human blood causing reduced S. aureus SH-*thyA* growth, biochemical complementation was achieved by the addition of 400 μg ml^−1^ thymidine. Plates were incubated aerobically at 37°C for 24 h.

Rather than an inhibitory factor in blood preventing the growth of the *pabA* mutant, it is possible that human blood leads to an increased requirement for dTMP, which cannot be met in a folate-deficient mutant reliant solely on the pyrimidine salvage pathway (Fig. 4a). Thymidylate synthase (encoded by *thyA*) is highly conserved, requiring THF as a cofactor for the conversion of dUMP to dTMP, an essential step in DNA synthesis. To maintain viability, *thyA* mutants can utilize extracellular thymidine via pyrimidine salvage pathways (31) and thus cannot grow *in vitro* on media lacking pyrimidines, such as Mueller-Hinton (MH) agar or human blood (27). To determine if human blood increases the demand for pyrimidines, a minimal permissive concentration of thymidine to allow *thyA* mutant growth (500 ng ml^−1^) was added to MH agar (Fig. 5a). As the concentration of added human blood increased, ranging from 1 to 50% (vol/vol), the growth of the *thyA* mutant was increasingly inhibited, suggesting that, as with the *pabA* mutant, pyrimidine requirements are elevated by human blood. This was further confirmed by the addition of a higher concentration of thymidine (400 μg ml^−1^), which allowed biochemical complementation of *thyA* (Fig. 5b).

In the host environment, when innate immune cells encounter bacteria, reactive oxygen species (ROS), such as superoxide and nitric oxide, are generated (32). Bacteria have developed sophisticated mechanisms to resist such oxidative stress. Although heme acquisition is a necessity for S. aureus survival *in vivo*, we hypothesized that heme causes oxidative stress on bacteria, increasing dTTP requirements for DNA repair, and that the *pabA* mutant would be less able to compensate than the parent strain. To test this hypothesis, the *pabA* mutation was transduced into a strain unable to acquire heme due to disrupted Isd and heme transport systems, LS1 Δ*isdE* Δ*htsA* (33). The triple mutant (LS1 Δ*isdE* Δ*htsA pabA*) was inoculated onto human blood agar in order to determine whether the removal of potential heme toxicity would restore the growth of the *pabA* mutant. No growth was observed for the *pabA* or Δ*isdE* Δ*htsA pabA* strain on unsupplemented blood agar, but both strains displayed good growth in the presence of exogenous pyrimidines (data not shown). However, it has been demonstrated that in the absence of functional heme transport and Isd systems, S. aureus can still acquire heme, by a third, as yet unknown heme transport mechanism (33).

### In the presence of sulfonamide antibiotics, nucleotide salvage pathways are required for S. aureus growth in blood.

The use of folate-antagonistic sulfonamide antibiotics, such as trimethoprim, against S. aureus leads to the loss of THF synthesis and, like the *pabA* mutation, a dependence on the pyrimidine nucleotide salvage pathway for dTMP synthesis. The activity of this class of antibiotics can be reversed by providing enough thymidine to bypass the requirement for the THF-dependent dTMP synthesis pathway (34). The reversal of trimethoprim activity against JE2, JE2-*pabA*, and JE2-*tdk* by pyrimidine was assessed by growth on human, sheep, or horse blood agar. On human blood, thymidine reversed the activity of trimethoprim against JE2, and JE2-*pabA* growth was restored in the presence of thymidine; however, trimethoprim was active against JE2-*tdk* in the presence or absence of thymidine (Table 3). Similar results were obtained on horse blood. On sheep blood, trimethoprim was inactive against JE2 and JE2-*pabA*, likely due to a higher pyrimidine concentration in sheep blood (35), demonstrating that the JE2-*pabA* phenotype on human blood may also be due to differences in the pyrimidine content of blood. As with human and horse blood, JE2-*tdk* was inhibited by trimethoprim on sheep blood, and the addition of thymidine could not reverse this inhibition, since the nucleotide salvage pathways are prohibited.

**TABLE 3:** Trimethoprim MICs for the S. aureus parent strain, JE2-*pabA*, and JE2-*tdk* on various media

Strain	Trimethoprim MIC (mg/liter) for the indicated strain on the following medium^*a*^:							
	BHI		Human blood		Sheep blood		Horse blood	
	−T	+T	−T	+T	−T	+T	−T	+T
Parent	1	>32	0.75	>32	>32	>32	1	>32
*pabA* mutant	1	>32		>32	>32	>32	>0.002	>32
*tdk* mutant	0.25	0.25	0.75	0.75	0.5	0.5	1	1

a −T, no exogenous thymidine added, +T, thymidine added at 400 μg ml^−1^.

## DISCUSSION

In order to identify novel pathogenicity determinants, an ordered library of transposon mutants was screened for gene disruptions causing growth and hemolysis defects on agar containing human blood as the only nutrient source. This screen identified *purA*, *purB*, and *pabA* as required for growth on human blood. The *purA* and *purB* genes are part of the *de novo* biosynthetic pathway for purines, and *pabA* is involved in folate synthesis. All three mutations were found to lead to significant attenuation in the zebrafish model of systemic infection, confirming the important role of these genes in pathogenesis.

In a study using a microarray-based system to detail the nonessential genes involved in the growth of Escherichia coli, Salmonella enterica, and Bacillus anthracis in human serum, the majority of mutants identified were involved in purine or pyrimidine biosynthesis (10). This suggests a scarcity of nucleotides *in vivo*, which bacteria counteract by being equipped with energetically costly metabolic pathways permitting *de novo* synthesis. Similarly, in our study, the ability of purine biosynthesis mutants to grow in nutrient-rich media suggested that *purA* and *purB* mutants require nutrients not readily available in human serum, whole blood, or the live zebrafish.

The reduced growth of S. aureus *pabA in vitro* was intriguing, because it was specific to human blood; normal growth was seen on blood components (serum, plasma), horse blood, and sheep blood. PabA is required for the production of PABA, an essential intermediate in the synthesis of THF. A *pabB* mutant of Streptococcus pneumoniae has been used as an attenuated strain for vaccine research, highlighting the importance of this pathway in the development of prophylactic strategies (36). The use of liquid CDM and solid CDM agar showed that purines, glycine, and serine were required for growth by the *pabA* mutant in excess of the amounts required by the parental strain. However, when assessed on human blood agar, growth inhibition could not be rescued with any compound except pyrimidines, suggesting that all other factors necessary to bypass the lack of THF are present in serum, plasma, and whole blood. The concept of “thymineless death” has been introduced previously and demonstrates the fundamental importance of pyrimidines in bacterial survival, over and above the other downstream effectors of THF (25). The addition of thymidine to human blood permitted the growth of the *pabA* mutant (Fig. S5a in the supplemental material). Human blood is known to have a lower thymidine content than the blood of other animals (35). However, the growth of the mutant on other thymidine-poor media (e.g., CDM, horse blood) suggested that thymidine deficiency *per se* was not solely responsible for the growth phenotype.

In the absence of THF-dependent *thyA* activity, pyrimidine salvage pathways are essential for the conversion of thymidine to dTMP (via Tdk), which is necessary for DNA replication. Both the *pabA* and the *thyA* mutant rely on these salvage pathways to provide a permissive amount of thymidine and, therefore, dTTP, allowing them to remain viable. It is difficult to tease apart exactly how human blood subverts this process, and we hypothesized that Tdk was the target of competitive inhibition in the *pabA* mutant, given that supplemental thymidine restored the growth of the *pabA* mutant, and a genetic knockout of *pabA* and *tdk* eliminated this biochemical complementation. Double knockouts of *pabA* with the gene responsible for the conversion of thymine to thymidine (*pabA pdp*) or with a pyrimidine transporter gene (*pabA nupC*) had no effect on biochemical complementation. Furthermore, the growth of the *pabA* mutant was reduced on human plasma supplemented with lysed RBC products. Since excess heme is toxic to S. aureus (28), heme and related molecules were ruled out as Tdk inhibitory factors. Tdk is a zinc-requiring enzyme that is purported to be required for transcriptional regulation (37). Zinc sequestration by human blood and other potential inhibitory factors should be investigated in future work (38).

Although the exact mechanism has yet to be elucidated, it is clear that human blood, or a component thereof, leads to an increased demand for dTMP, which cannot be met in a THF-deficient mutant; hence, exogenous thymidine or thymine is necessary to support the growth of the mutant specifically on human blood.

Finally, little is known about the clinical prevalence or relevance of *pabA* mutations. Trimethoprim is used in the control of S. aureus infections, and long-standing treatment can lead to failure due to the development of antibiotic resistance (39). In this context, *thyA* mutations are usually observed in the resistant subpopulation, and such mutations cause the formation of thymidine-dependent small-colony variants (SCVs), which rely on pyrimidine salvage pathways (via Pdp and Tdk) (40). However, sulfonamide antibiotics remain bactericidal unless a thymidine-rich environment exists, such as damaged host tissues, which allow S. aureus to utilize pyrimidine salvage pathways and thus survive (41). The work presented here suggests that the activity of sulfonamide drugs results from the inhibition of THF coupled with reduced activity of the pyrimidine salvage pathways and/or an increased demand for dTMP imparted by human blood. The identification of metabolic pathways important for host-pathogen interactions provides novel avenues to be explored in the effort to combat antibiotic-resistant pathogens.

## MATERIALS AND METHODS

### Ethics statement.

Zebrafish embryos less than 5 days postfertilization (dpf) are not protected under the Animals (Scientific Procedures) Act 1986, but all zebrafish work was carried out according to the details set out in Project License PPL 40/3574. Murine work was carried out according to UK law in the Animals (Scientific Procedures) Act 1986, under Project License PPL 40/3699. Human blood was obtained from healthy volunteers in compliance with the guidelines of the South Sheffield Research Ethics Committee (STH13927).

### Bacterial strains, plasmids, and growth conditions.

The Nebraska transposon mutagenesis library (11) was acquired from the Network on Antimicrobial Resistance in S. aureus (NARSA) strain repository, now available from BEI Resources (www.beiresources.org/), and was used for screening experiments. Originally in the USA300 LAC JE2 background, mutations were transduced back into JE2 or SH1000 as required. All other strains, and the plasmids used in this study, are listed in Table 4. S. aureus strains were routinely grown in brain heart infusion (BHI) medium at 37°C with aeration at 250 rpm, unless otherwise stated. Mueller-Hinton agar (Oxoid) was used as a thymidine-poor medium where stated. E. coli strains were grown in Luria-Bertani medium at 37°C with aeration at 250 rpm. Agar at 1.5% (wt/vol) was added for solid media. Antibiotics were added as required. For MIC determination, a bacterial colony was inoculated into 2 ml sterile distilled water (dH_2_O) and was spread onto an agar plate using a sterile swab (Oxoid). Trimethoprim Etests (bioMérieux) were applied to the solid medium surface using tweezers, and incubation was carried out overnight at 37°C.

**TABLE 4:** Strains and plasmids used in this study

Strain or plasmid	Relevant genotype/markers	Source or reference
Strains		
S. aureus strains		
SH1000	Functional *rsbU*^+^ derivative of 8325-4	51
RN4220	Restriction-negative, modification-positive strain	52
USA300 JE2	USA300 LAC strain cured of plasmids p01 and p03	11
SJF4669	SH-*pabA*::*spc pdp*::*ery*	This study
SJF4670	SH-*pabA*::*spc nupC*::*ery*	This study
SJF4671	SH-*thyA*::*ery*	27
SJF4678	SH-*pabA*::*spc tdk*::*ery*	This study
JC006	JE2-*pabA*, pJC002 inserted at lipase; *pabA*^+^ Ery^r^ Lin^r^ Tet^r^	This study
JC007	JE2-*pabA*, pKASBAR inserted at lipase; Ery^r^ Lin^r^ Tet^r^	This study
JC010	SH-*pabA*, pJC002 inserted at lipase; *pabA*^+^ Ery^r^ Lin^r^ Tet^r^	This study
JC011	SH-*pabA*, pKASBAR inserted at lipase; Ery^r^ Lin^r^ Tet^r^	This study
LS1	Spontaneous murine arthritis isolate	53
LS1 Δ*isdE* Δ*htsA*	LS1 derivative; Δ*isdE* Δ*htsA*	33
E. coli TOP10	F^−^ *mcrA* Δ(*mrr-hsdRMS-mcrBC*) ϕ80 *lacZ*ΔM15 Δ*lacX74 recA1 deoR araD139* Δ(*ara-leu*)*7697 galK rpsL* (Str^r^) *endA1 nupG*	Invitrogen
Plasmids		
pKASBAR	Hybrid vector of pCL84 and pUC18 for integration into S. aureus lipase gene (*geh*), *attP*; Tet^r^ (S. aureus); Spec^r^ (E. coli)	46
pJC002	pKASBAR containing the *pab* operon, with *pabA*, *pabB*, and *pabC*, and upstream control elements; Tet^r^	This study

The chemically defined medium used in this study has been described previously (42). The following components were dissolved in 1 liter of H_2_O: Na_2_HPO_4_·2H_2_O, 7 g; KH_2_PO_4_, 3 g; l-aspartic acid, 0.15 g; l-alanine, 0.1 g; l-arginine, 0.1 g; l-cysteine, 0.05 g; glycine, 0.1 g; l-glutamic acid, 0.15 g; l-histidine, 0.1 g; l-isoleucine, 0.15 g; l-lysine, 0.1 g; l-leucine, 0.15 g; l-methionine, 0.1 g; l-phenylalanine, 0.1 g; l-proline, 0.15 g; l-serine, 0.1 g; l-threonine, 0.15 g; l-tryptophan, 0.1 g; l-tyrosine, 0.1 g; l-valine, 0.15 g; biotin, 0.02 g; pyridoxal HCl, 0.8 g; nicotinic acid, 0.4 g; pyridoxamine di-HCl, 0.8 g; d-pantothenic acid, 0.4 g; riboflavin, 0.4 g; thiamine HCl, 0.4 g; adenine sulfate, 0.02 g; guanine HCl, 0.02 g; CaCl_2_·6H_2_O, 0.01 g; (NH_4_)_2_Fe(SO_4_)_2_·6H_2_O, 0.006 g; glucose, 10 g; MgSO_4_·7H_2_O, 0.5g. Inosine was used at 20 µg/ml.

Human, or other animal, blood or blood components were added to agar at various concentrations, as required. Venous blood was collected from healthy volunteers following informed consent. For plasma preparation, blood was centrifuged at 270 × *g* for 20 min in 50-ml Falcon tubes. The upper, platelet-rich phase was collected and was either used directly as platelet-rich plasma (PRP) or centrifuged again at 1,155 × g for 30 min to yield platelet-poor plasma (PPP). Plasma was stored at −20°C. Animal blood and blood products were purchased from Thermo Scientific or Sigma and were stored at 4°C. Bovine hemin or hemoglobin, human hemoglobin, thymine, thymidine, glycine, serine, PABA, or folic acid was added to media as and when required.

### Genetic manipulation.

Electroporation was used to transform S. aureus RN4220 and E. coli using previously published methods (43, 44). All S. aureus transduction experiments were carried out with ϕ11 as described previously (45).

For genetic complementation of SH-*pabA* and JE2-*pabA*, Phusion polymerase (NEB) was used to amplify the *pab* operon from S. aureus SH1000 genomic DNA, using primers containing appropriate restriction sites (forward, ATAATAGGGCCCATTGTA-CTGTCTTGACCACCACT; reverse, ATAATACTCGAGATACGTATACAAGAATTAA-CAACAGCA). The PCR product was inserted into pKASBAR (46), a plasmid encoding an *attP* site. Using this *attP* site, bacteriophage DNA can integrate into the S. aureus genome at the *attB* site, in the presence of an integrase (47). The *attB* site is located at the glycerol ester hydrolase (*geh*) gene, so integration can be verified by loss of lipase activity. For such genetic manipulation, the integrase is provided by an additional helper plasmid, pYL112Δ19, propagated in the S. aureus recipient strain, RN4220. The insert was then transduced from RN4220 into *pabA* and control strains.

To prepare double mutants within Tn insertions, the “toolkit” for switching antibiotic resistance within NTML strains was used as published previously (48). Tn inserts in *pdp*, *nupC*, and *tdk* genes, with alternate antibiotic resistance markers, were transduced into the *pabA* mutant as listed in Table 4.

Strains LS1 and LS1 Δ*isdE* Δ*htsA* were kindly provided by Sean Nair (University College London). *pabA* was transduced into both strains, and successful transductants were confirmed by PCR.

### Transposon library screen.

The NTML was grown for 18 h at 37°C in 96-well microtiter dishes. Using a 96-pin replicator (Boekel Industries), the contents of each well were transferred to BHI agar, BHI agar plus erythromycin (10 μg/ml) and lincomycin (25 μg/ml), 30% (vol/vol) human blood agar, 50% (vol/vol) bovine serum agar, and 5% (vol/vol) sheep blood, plus Columbia agar base in rectangular OmniTray plates (Nunc). Human blood and bovine serum plates were incubated for 48 h at 37°C; all other plates were incubated for 18 h at 37°C, with an additional 4 h at 4°C for sheep blood plates, to ensure efficient hemolysis. Phenotypes were determined by comparison of each spot (colony size and hemolysis zone) to the surrounding spots on the plate.

### Zebrafish model.

Zebrafish embryos, strain London wild type (LWT), were maintained in E3 medium at 28°C by following standard protocols (17). Embryos were bred in the aquarium facilities at the University of Sheffield. Microinjection of embryos was performed as described previously (17). Individual infected embryos were kept in 100 μl E3 medium, and survival was assessed over 90 h. For *in vivo* complementation experiments, compounds were dissolved in E3 medium and were buffered to a pH of 6.5 to 7.5. Immediately following injections, embryos were placed in appropriate compound solutions. Further compound solution was added in the embryo washing step. Ninety-six-well microtiter plates were placed in a plastic box, with damp paper, to reduce evaporation during incubation.

*pu.1*-antisense morpholino-modified oligonucleotides (49) were injected into zebrafish embryos using the method described previously (17). Bacteria were recovered from infected embryos at 12-h intervals. Individual embryos were transferred to microcentrifuge tubes and were homogenized using a PreCellys 24-Dual homogenizer (Peqlab). Bacterial numbers were then determined by serial dilution in phosphate-buffered saline (PBS) and plating onto BHI agar.

### Murine infection model.

Female BALB/c mice were purchased from Charles River Laboratories (Margate, UK) and were maintained by standard husbandry techniques at the University of Sheffield (Biological Services). Bacteria were washed in endotoxin-free PBS (Sigma), and 100 μl (2 × 10^7^ to 4 × 10^7^ CFU) was injected intravenously (i.v.) into the tail vein. Serial dilutions of culture were prepared to confirm the injection CFU. Mice were monitored and were sacrificed at 72 hpi. Mouse organs were individually homogenized in PBS and, after serial dilution, plated onto BHI agar supplemented with antibiotics as needed for bacterial enumeration.

### Statistical analysis.

Sample sizes were predetermined for mouse (*n* = 10) and zebrafish (*n* = 20) experiments based on previous experimental data (50). All zebrafish experimental results are representative of 2 experiments unless otherwise stated. For zebrafish embryo survival experiments, the Kaplan-Meier method was employed. Survival curves were compared using the log rank (Mantel-Cox) test. For bacterial count comparisons in murine experiments, the Mann-Whitney U test was used. Statistical analysis was performed using Prism, version 6.0 (GraphPad), and a *P* value of <0.05 was considered significant.

## Supplementary Material

Supplemental material
