# Supplementary material for: Identification of Staphylococcus aureus Factors Required for Pathogenicity and Growth in Human Blood
Source: Infect Immun. 2017 Oct 18;85(11):e00337-17. doi: 10.1128/IAI.00337-17 (PMC5649012; doi:10.1128/IAI.00337-17)
Supplement: Supplemental material [file supp_85_11_e00337-17__index.html]

Supplemental material 

# Identification of Staphylococcus aureus Factors Required for Pathogenicity and Growth in Human Blood

## Supplemental material

- Supplemental file 1 -

  Fig. S1. Virulence of *S. aureus* strains displaying altered hemolysis and bacterial numbers of the parent strain in the zebrafish model of infection. Fig. S2. Purine *de novo* biosynthesis pathway. Fig. S3. Growth of *pabA* on human blood/blood component agar. Fig. S4. Metabolic functions of folate. Fig. S5. Role of PabA in the nutritional requirements of *S. aureus*.

  PDF, 5.4M
