# Supplementary material for: Identification of Staphylococcus aureus Factors Required for Pathogenicity and Growth in Human Blood
Source: Infect Immun. 2017 Oct 18;85(11):e00337-17. doi: 10.1128/IAI.00337-17 (PMC5649012; doi:10.1128/IAI.00337-17)
Supplement: Supplemental material [file IAI.00337-17_zii999092187s1.pdf]

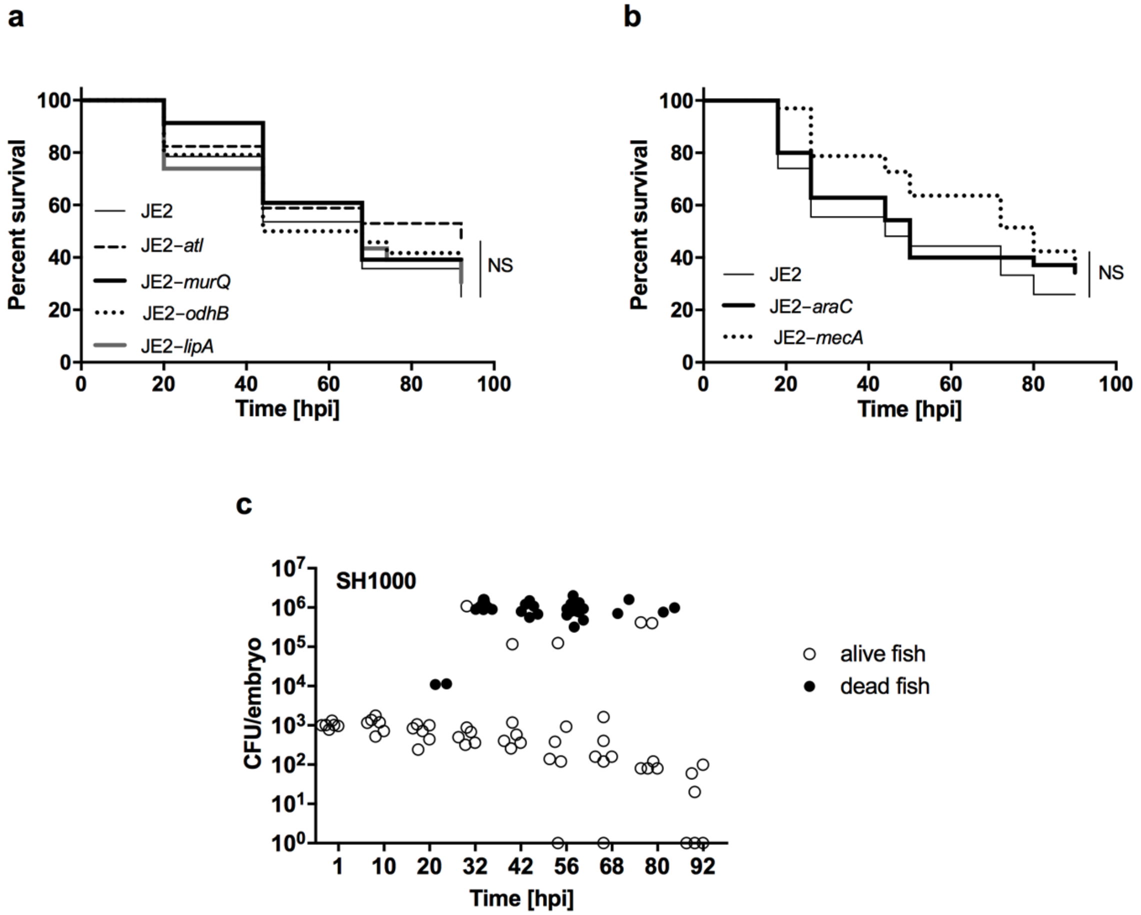

Figure S1 Virulence of *S. aureus* strains displaying altered haemolysis and bacterial numbers of the parent strain, in the zebrafish model of infection

a,b, Survival curves of fish injected with *S. aureus* JE2 (1500 CFU) or *S. aureus* JE2 mutants identified in the Tn library screen showing altered haemolysis on human blood agar. NS, not significant. **c,** Growth of *S. aureus* SH1000 within embryos after injection with 1500 CFU. Open circles, live and filled circles, dead embryos.

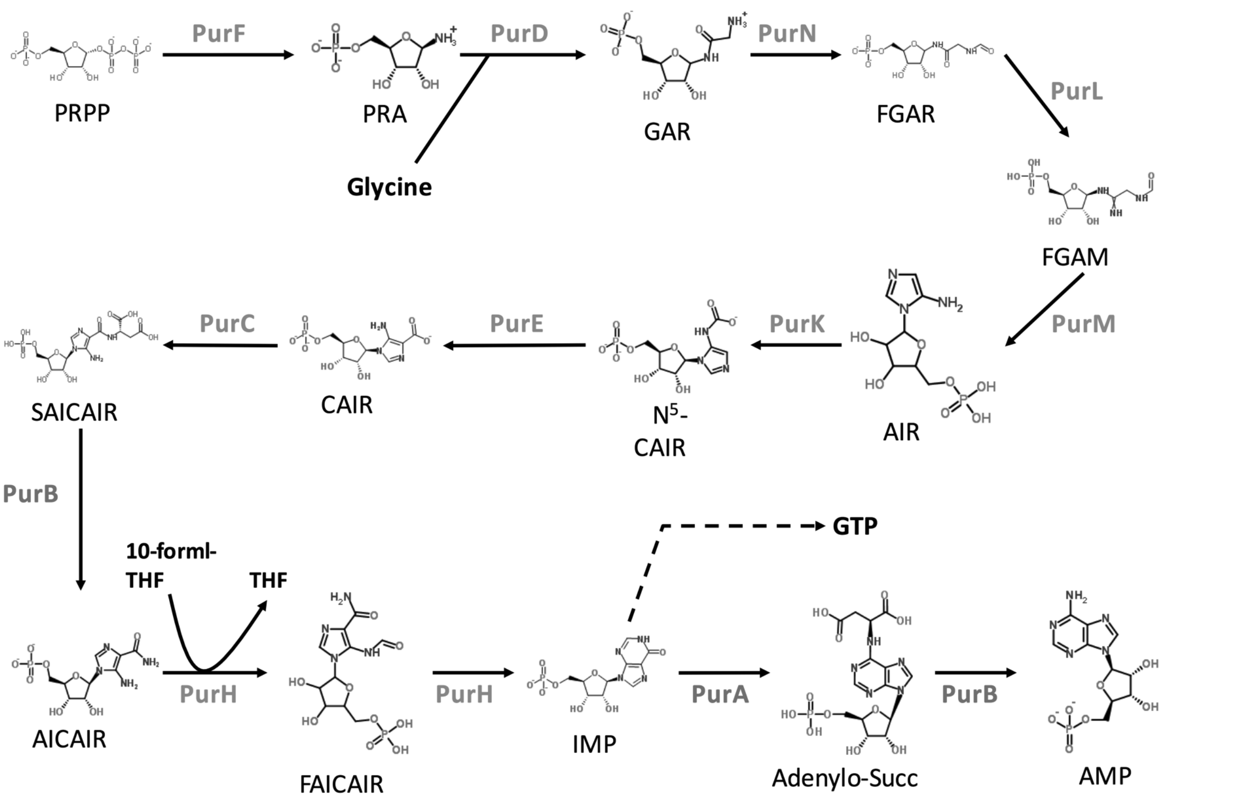

Figure S2 The purine *de novo* biosynthesis pathway

PurB has two roles in the pathway, one before IMP branching and one after branching leading to ATP synthesis (1–3). Abbreviations: PRPP, 5-phospho-α-D-ribose 1-diphosphate; PRA, 5-phospho-β-D-ribosylamine; GAR, N1-(5-phospho-β-D- ribosyl)glycinamide; FGAR, N2-formyl-N1-(5-phospho-β-D-ribosyl)glycinamide; FGAM, 2-(formamido)-N1-(5-phospho-β-D-ribosyl)acetamidine; AIR, 5-amino-1-(5- phospho-β-D-ribosyl)imidazole; N5-CAIR, N5-carboxyaminoimidazole ribonucleotide; CAIR, 5-amino-1-(5-phospho-D-ribosyl)imidazole-4-carboxylate; SAICAIR, 5'- phosphoribosyl-4-(N-succinocarboxamide)-5-aminoimidazole; AICAIR, 5-amino-1-(5-phospho-D-ribosyl)imidazole-4-carboxamide; FAICAIR, 5-formamido-

1-(5- phospho-D-ribosyl)-imidazole-4-carboxamide; IMP, inosine monophosphate, Adenylo-succ, N6-(1,2-dicarboxyethyl)-AMP; AMP, adenosine-monophosphate.

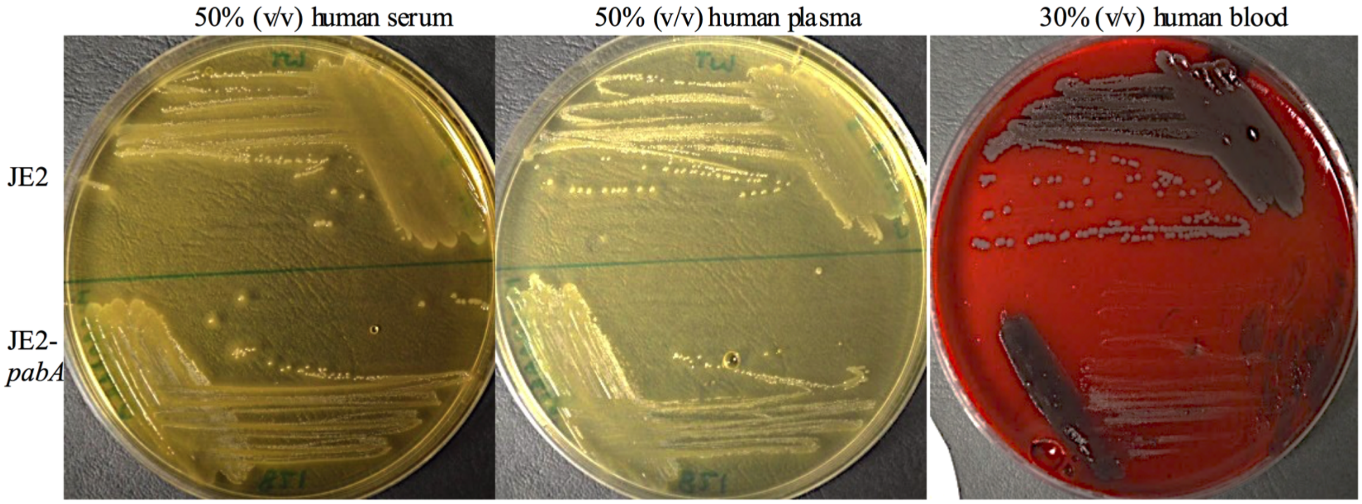

Figure S3 The growth of *pabA* on human blood/blood component agar

Growth of parent (JE2) or *pabA* on human serum (50% v/v), human plasma (50% v/v) or whole human blood (30% v/v) agar. Plates were incubated aerobically at 37°C for 48 h.

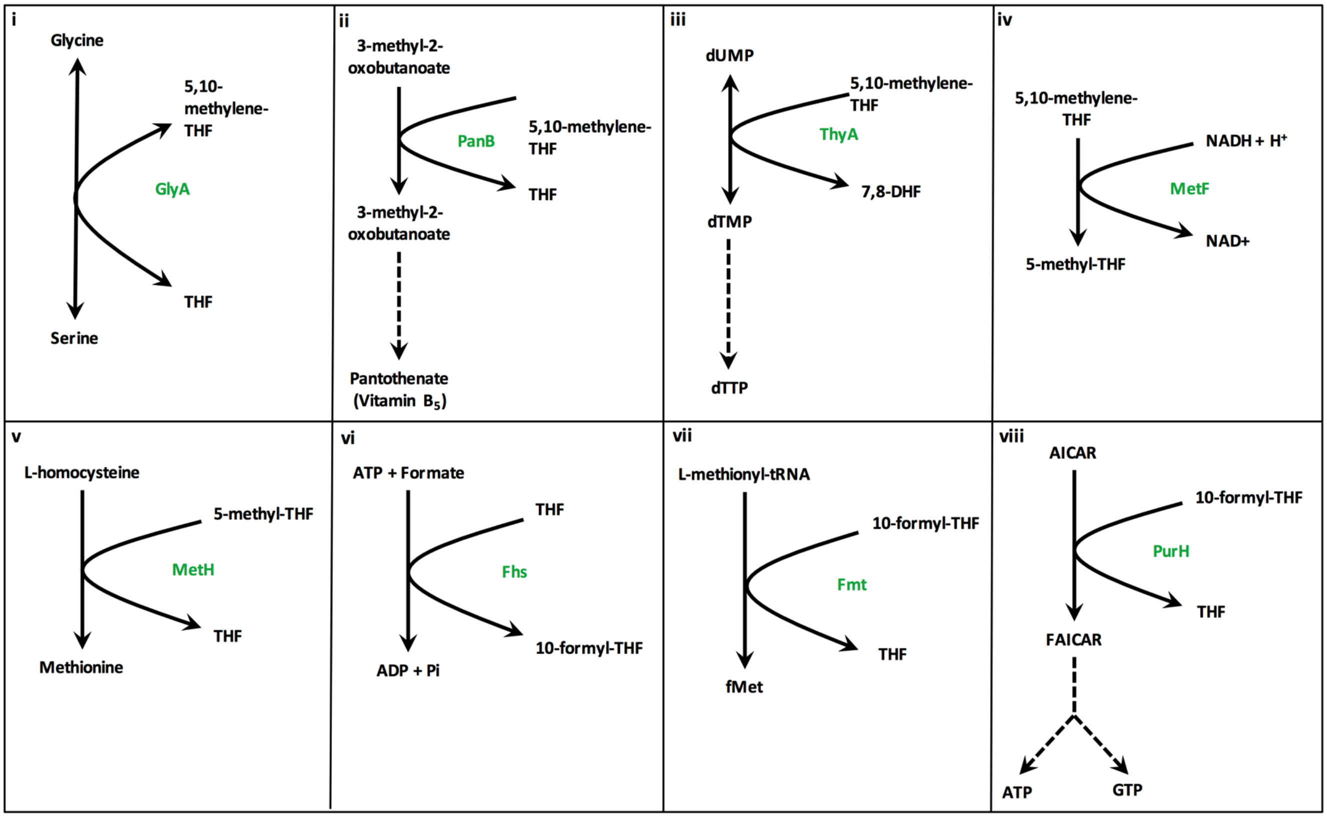

Figure S4 The metabolic functions of folate

Enzymes using folate as a cofactor are shown. **i,** serine/glycine interconversion; **ii,** vitamin B5 biosynthesis; **iii,** synthesis of dTTP from dTMP **iv,** conversion of 5,10-methylene-THF to 5-methyl-THF; **v,** remethylation of L-homocysteine; **vi,** 10-formyl-THF production from THF; **vii,** use of 10-formyl-THF to formylate L-methionine charged tRNA; **viii,** 10-formyl-THF-dependent conversion of AICAIR to FAICAIR (purine *de novo* biosynthesis).

Dashed lines - 1 or more steps not shown. (1)

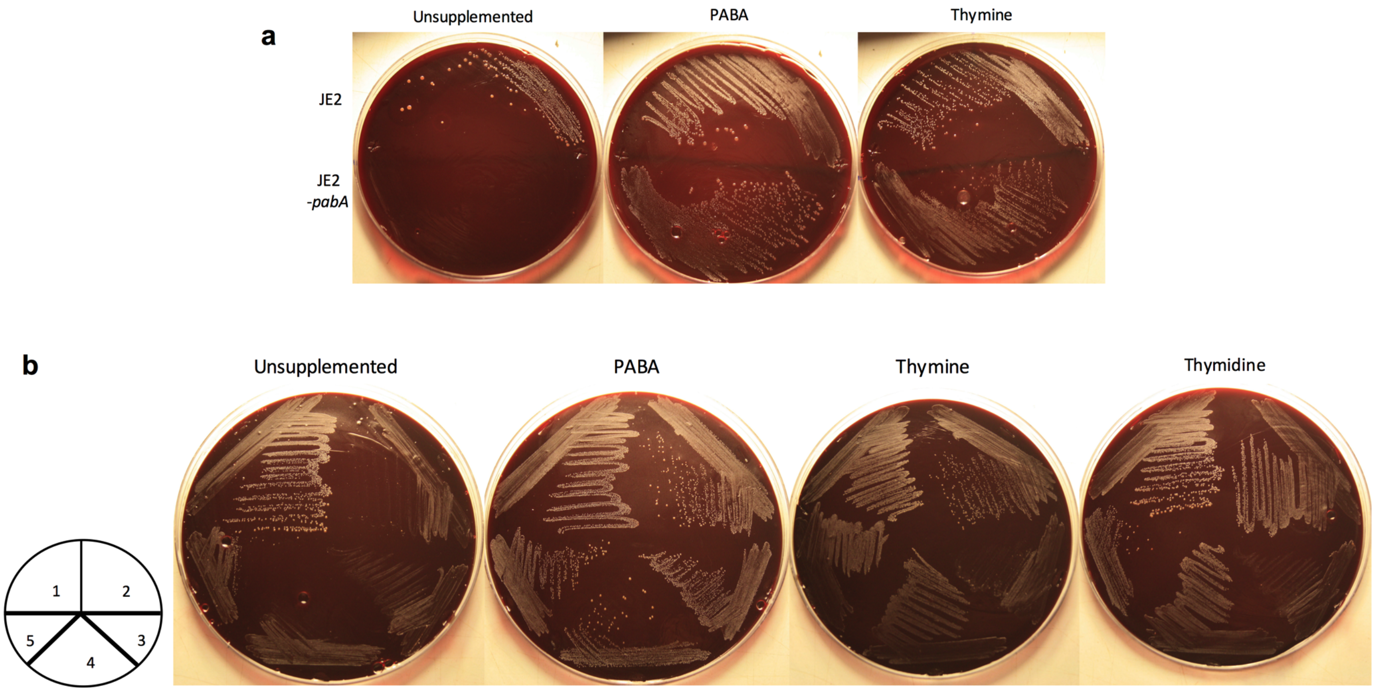

Figure S5 Role of PabA in the nutritional requirements of *S. aureus*

a, Growth of *S. aureus* JE2 and JE2-*pabA* on human blood agar (30% v/v) either unsupplemented or supplemented with PABA (20 µg ml^{-1}) or thymine (400 µg ml^{-1}). **b,** Growth of *S. aureus* SH1000 (1), *pabA*^{spc} (2), *pabA*^{spc}*tdk*^{ery} (3), *pabA*^{spc}*pdp*^{ery} (4), or *pabA*^{spc}*nupC*^{ery} (5) on human blood agar (30% v/v) either unsupplemented or supplemented with PABA (20 µg ml^{-1}); thymine (400 µg ml^{-1}) or thymidine (400 µg ml^{-1}). Key to strain positions is as shown.

References

1. Caspi R, Altman T, Billington R, Dreher K, Foerster H, Fulcher CA, Holland TA, Keseler IM, Kothari A, Kubo A, Krummenacker M, Latendresse M, Mueller LA, Ong Q, Paley S, Subhraveti P, Weaver DS, Weerasinghe D, Zhang P, Karp PD. 2014. The MetaCyc database of metabolic pathways and enzymes and the BioCyc collection of Pathway/Genome Databases. Nucleic Acids Res 42:D459–D471.

2. Kanehisa M, Goto S. 2000. KEGG: kyoto encyclopedia of genes and genomes. Nucleic Acids Res 28:27–30.

3. Pence HE, Williams A. 2010. ChemSpider: An Online Chemical Information Resource. J Chem Educ 87:1123–1124.
